# What Else Can CD39 Tell Us?

**DOI:** 10.3389/fimmu.2017.00727

**Published:** 2017-06-22

**Authors:** Hai Zhao, Cong Bo, Yan Kang, Hong Li

**Affiliations:** ^1^Department of Critical Care Medicine, West China Hospital, Sichuan University, Chengdu, China; ^2^Key Laboratory of Obstetrics & Gynecology, Pediatric Diseases, and Birth Defects of the Ministry of Education, West China Second Hospital, Sichuan University, Chengdu, China

**Keywords:** CD39, extracellular ATP, adenosine, Tregs, γδ T cell, CD161, Th17 cell, Bregs

## Abstract

As the rate-limiting enzyme in ATP/ADP–AMP–adenosine pathway, CD39 would be a novel checkpoint inhibitor target in preventing adenosine-triggered immune-suppressive effect. In addition, CD39^hi^ Tregs, but not CD25^hi^ Tregs, exhibit sustained Foxp3 levels and functional abilities, indicating it could represent a new specific marker of Tregs. Similarly, inhibition of CD39 enzymatic function at the surface of tumor cells alleviates their immunosuppressive activity. Far from conclusive, present research revealed that CD39 also dephosphorylated and thus inactivated self- and pathogen-associated phosphoantigens of Vγ9Vδ2 T cells, which may be the most promising subpopulation for cellular vaccine. CD39 is also tightly related to Th17 cells and can be regarded as a Th17 cells marker. In this review, we focus on present research of CD39 ectoenzyme and provide insights into its clinical application.

## Introduction

Extracellular adenosine and ATP exert important functions in physiology and pathophysiology. They for instance play key role in heart and vascular function, during pregnancy (1) and in immune responses (2). Outside the cell, extracellular ATP (eATP) acts as danger-associated molecular patterns (DAMPs) and can bind to purinergic receptors to trigger signaling cascades to induce an inflammatory response (3). However, adenosine is a potent immune-suppressor of cells that express A2 and A3 receptors, such as lymphocytes (4). To avoid ATP-induced pathological effects, ATP can be hydrolyzed into adenosine and phosphate by a cascade of enzymes, of which CD39 is the most important.

CD39, the NTPDase (ecto-nucleoside triphosphate diphosphohydrolase), regulates immune responses balance by hydrolyzing ATP and ADP. It is now again becoming a newly recognized “immune checkpoint mediator” that interferes with antitumor or anti-inflammatory immune response (5, 6). Moreover, some recent research has revealed a number of neoteric functions of CD39, which display its close relation with Tregs (7–9), Th17 cells (10, 11), γδ T cells (12), and Bregs (13). It follows from this reasoning that CD39 acts as a key molecule in inflammation (14–16) and tumor immunity (17–20).

In the present review, we will discuss the current knowledge on the role of CD39 expressed on different types of cells and explore its potential in inflammation and tumor immunity.

## Classic Features of CD39

Cellular ATP serves as the main energy currency, driving virtually all cell functions. Self-evidently, intracellular ATP plays important pathophysiological roles. In terms of eATP, it is ubiquitously used for cell–cell communication in physical setting. Low concentration of eATP-detected surrounding resting cells indicates the presence of neighboring living cells, especially in nervous (21) and vascular systems (22). And also, this biochemical substance can be poured into extracellular space upon, for instance, tissue stress such as necrosis, apoptosis, hypoxia, or inflammation (23). Release from activated or apoptotic cells is done *via* two mechanisms as follows: transport *via* membrane-bound channels or transporters and exocytosis of intracellular vesicles (24). Additional work showed that kindred purinergic signaling pathways regulate critical aspects of many other physiological processes, including immune response (25).

Recent years, the role of eATP in immune system has broaden our horizon. ATP release in response to inflammatory mediators is a basic mechanism required for neutrophil activation and immune defense (26). The steady-state cytosolic concentration of ATP is 3–10 mM, whereas eATP is only~10 nM (27), which is maintained as a result of the activities of extracellular ecto-apyrases and CD39. The enzymatic activities of CD39 and CD73 play paramount roles in calibrating the particularity, duration, and magnitude of purinergic signals *via* the conversion of ATP/ADP to AMP and AMP to adenosine, respectively. The ATP-CD39–CD73–adenosine cascade is strictly controlled by enzymatic activity, in which CD39 serves as the rate-limiting enzyme (28).

**Figure 1:**
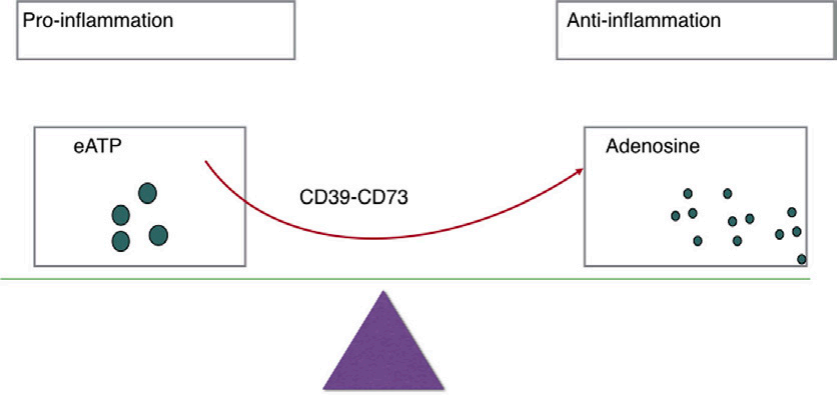
Ectoenzymes, e.g., CD39, CD73 mediate the metabolization of extracellular ATP (eATP) to adenosine. eATP signals through P2X and P2Y purinergic receptors to induce inflammation while adenosine exerts immunosuppressive activity on immune cells and thereby protects tissues against excessive inflammation.

Balance between eATP and adenosine (see Figure 1) is crucial in immune homeostasis since eATP is a danger signal released by injured or apoptosis cells that acts to prime immune responses through the ligation of P2 receptors (2). There are two subsets of P2 receptors: P2X or P2Y receptors (23). Seven P2X receptors plus eight P2Y receptors have been identified in humans (26, 29). Responses to low eATP are mediated by P2 receptors with high or intermediate affinity for eATP (EC50 <20 µM), while responses to high eATP are mediated by P2X7 (EC50 >100 μM) (27). As for a further explanation, eATP functions as DAMPs and then binds to P2 receptors, resulting in heightened inflammation and regulatory cell inhibition in most cases (30).

ATP has varieties of pro-inflammatory effects. Since sundry immune cells express most of the ATP receptors, ATP can affect most immune cells. For example, eATP released by damaged cells can activate the immune system *via* the stimulation of P2X7 receptors on DCs and then promote the secretion of IL-1β and IL-18 (31). Next, IL-1β will facilitate macrophages maturation and their cytokine production increase (32). Similarly, IL-18 would boost NK cells proliferation and strengthen IFN-γ production plus cytotoxicity (33). While in T cells, ATP activates T cells by inducing IL-2 production and cytotoxicity. Moreover, it induces differentiation toward pro-inflammatory Th17 cells, while it inhibits the differentiation toward Tregs. From a micro perspective, eATP stimulates Ca^2+^ entry through P2X purinergic receptor channels and Ca^2+^ mobilization to facilitate Ca^2+^-calmodulin-dependent activation and nuclear translocation of nuclear factor of activated T cells (NFAT), which stimulates the production of IL-2, pannexin 1 channels, and other NFAT targets. Autocrine ATP release helps to sustain P2 purinergic receptor signaling and NFAT activation (34).

Adenosine—the counterpart of ATP, which is produced by breakdown of ATP—is nothing of a novelty. As early as 1980s, adenosine has been used to slow down the heart rate of patients suffering from supraventricular tachycardia (22). As for immune system, rather than activating T cells responses, adenosine conversely inhibits T cells responses including Tregs and Th cells. But it is not contradictory that adenosine inhibits the differentiation toward Th17 cells while it promotes differentiation toward Tregs. It is actually regarded as a key endogenous molecule that regulates tissue function by activating four G-protein-coupled P1 receptors, denoted A1, A2A, A2B, and A3 (35). A1 and A2A are high-affinity receptors, while A2B and A3 are low-affinity receptors (36). Meanwhile, A2A and A2B stimulate adenylyl cyclase, while A1 and A3 inhibit adenylyl cyclase (36). P1 receptors are expressed on kinds of immune cells such as macrophages, dendritic cells, and lymphocytes. There are now varieties of promising emerging therapeutic approaches centered on the modulation of adenosine in the immune system (37). Triggering different receptors can have different consequence. Importantly, A2A receptors are closely related to cyclic adenosine monophosphate (cAMP) response element-binding protein, which eventually leads to the transcription of the CEBPβ gene (38). While CEBPβ protein binds to the IL-10 gene promoter, which triggers IL-10 transcription, and subsequently leads to the release of IL-10 (38). IL-10, human cytokine synthesis inhibitory factor, was reported to suppress cytokine secretion, antigen presentation, and CD4^+^ T cell activation (39). One interesting research to mention is that resilient individuals seem to have a better anti-inflammatory response compared to posttraumatic stress disorder patients since they present higher IL-10 levels (40).

The ultimate effect of ATP and adenosine during immune responses depends on the balance between the two molecules. Of note, though CD39 and CD73 expressed on immune cells decrease local ATP levels while increase local adenosine levels, the substrate and catalytic product of ATP/ADP–CD39–AMP–adenosine pathway, are not actually simple yin and yang in immune responses (41). In fact, both ATP and adenosine may have dual effects on immune responses, depending on concentration, the duration of the exposure, and the conditions of the *in vivo* environment. Under pronged exposure or at low concentrations, as mentioned before, responses to eATP are mediated by P2R with EC50 <20 μM (41). Then, it will reduce secretion of inflammatory cytokine including IL-1β, IL-6, IL-12, and TNF-α, etc., of macrophages or mature DCs (42).

## CD39 Expressed on Different Populations of Immune Cells

### CD39 and Tregs

Tregs play an indispensable role in maintaining immunological unresponsiveness to self-antigens and in suppressing excessive immune responses deleterious to the host. In order to better explain the relationship between CD39 and Tregs, it is necessary to make sense of how Tregs work?

We must realize that there is still a long way to figure out the comprehensive mechanism of Tregs. The molecular mechanisms of suppression remain incompletely understood and even all the present conclusions are not agreed at all. A variety of molecules are involved in Treg-mediated suppression mechanisms, including IL-2, cytotoxic T-lymphocyte-associated protein 4 (CTLA-4), glucocorticoid induced tumor necrosis factor receptor (GITR), IL-10, TGF-β, IL-35, LAG3 (lymphocyte-activation gene3), granzyme B, adenosine, and cAMP (43). Given that ectopic Foxp3 expression in conventional T cells can function Treg-like suppressive activity, the molecule(s) mediating a core suppressive mechanism may well be controlled by Foxp3 (44–46). Foxp3 indeed dominates the expression of the above molecules and deficiencies of these molecules would produce similar autoimmune diseases as observed in Foxp3 deficiency (43, 47). We are not going to discuss the intricate mechanisms here in detail, but we are glad to introduce some recent research on the progress.

**Figure 2:**
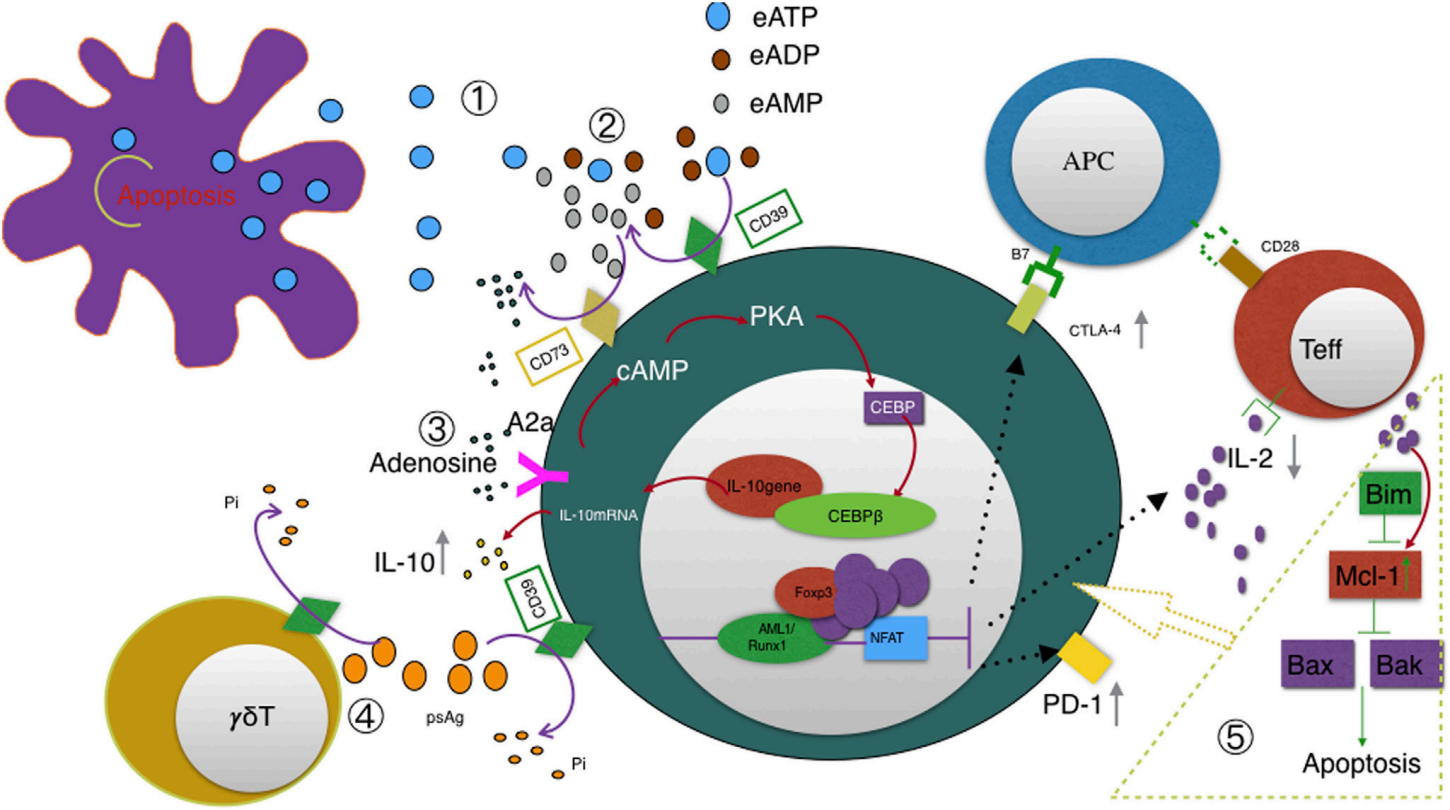
Illustration of CD39 function ① eATP accumulates in the extracellular space in response to metabolic stress or cell damage such as apoptosis. ② CD39 initiates extracellular adenosine generation by catalyzing the degradation of ATP and ADP to AMP; CD73 also has ecto-5′-nucleotidase enzyme activity that catalyzes the dephosphorylation of AMP to adenosine; CD39, not CD73, is the rate-limiting enzyme of the cascade leading to the generation of suppressive adenosine. ③ Adenosine activates A2A receptor and subsequently triggers pathways converge on CEBPβ to induce IL10 production. ④ CD39 also dephosphorylates pAgs of Vγ9Vδ2 T cells. This degradation may also be catalyzed by CD39 expressed on Tregs and possibly represents a novel mechanism of Tregs suppressing Vγ9Vδ2 T cells. CD39 upregulation acts as a feedback mechanism to desensitize Vγ9Vδ2 T cells to self- and pathogen-associated pAgs. ⑤ Pro-apoptotic Bim, antiapoptotic Mcl-1, and apoptotic regulators Bax and Bak altogether contribute to T cells homeostasis and survival. Especially, IL-2 and costimulatory signals upregulate Mcl-1 expression and hence allows Tregs to proliferate. We speculate that CD39 is involved in the above signal transduction since CD39 were reported to be associated with T cells apoptosis.

There has been some research focused on apoptotic mechanism of Tregs recently. It was found that Tregs proliferate more rapidly than other CD4^+^CD25^−^ conventional T cells under a static condition (48). Mcl-1, a member of Bcl-2 protein family (49, 50), is the dominant antiapoptotic in maintaining their dynamic changes—Tregs will proliferate after IL-2 elevates antiapoptotic Mcl-1 expression. Mcl-1 in turn inhibits Bax-mediated intrinsic apoptotic pathway and hence allows Tregs to proliferate in another way (48). Moreover, Fang et al. reported that CD39 not only can identify human CD4^+^ T cells prone to apoptosis but it is more readily induced in CD4^+^ T cell response of elder individual (51). CD39 may also play a key role in Tregs homeostatic balance and the relationship between CD39 and apoptosis deserves further study (see Figure 2).

CD39 has been reported to be found on the surface of human and murine naive Tregs (52, 53). The ATP–CD39–CD73–adenosine axis contributes to Foxp3^+^ CD4^+^ suppressor T cell activity (54). A subset of CD4^+^ T cells express CD39 combined with CD25, GITR, and CTLA-4 molecules, which are commonly found on Foxp3^+^CD4^+^ T cells (55). More importantly, CD39^+^ CD4^+^T cells do express Foxp3. Indeed, A2A receptors (the main adenosine receptors) stimulation inhibits IL-6 expression while promoting TGF-β production (56). Antigenic stimulation of naïve T cells in the presence of TGF-β leads to FOXP3 expression, and consequently, Tregs function (57). Thus, upon binding to A2A receptors, adenosine not only suppresses effector T cells functions but also promotes the induction of adaptive Tregs.

Interestingly, CD39 has so far only been known as an ecto-ATPase. But, Gruenbacher et al. proved that CD39 also dephosphorylates and thus inactivates self- and pathogen-associated phosphoantigens (12). We speculate that hydrolyzing specific antigens would also be another option by which Tregs functions, at least for Vg9Vd2 T cells. An experiment designed to confirm this mechanism of Treg is now being conducted in our laboratory.

At this stage, Tregs are still purified dependent on the expression level of CD25—CD4^+^CD25^+^, CD4^+^CD25^high^, or CD4^+^CD25^high^ FOXP3^+^. Considering that CD25 (IL-2Rα) is also widely expressed on effector lymphocyte besides Treg, it is not an ideal surface marker actually. It has been confirmed that CD39 is predominantly expressed on human CD4^+^ Foxp3^+^ T cells, and that its expression level is proportional to the Foxp3 expression level (52). Thus, CD39 can be a competent surface marker for routine isolation of functionally active human Tregs from the peripheral blood of healthy donors or patients. In addition, CD39 expression is considered null in Foxp3^+^CD4^+^ T cell development since CD39-deficient mice still possess peripheral CD4^+^CD25^+^ T cells. But the conclusion has limitations because CD39-knockout may just induced another equally critical detour pathway during Treg development and maybe both of them cannot work at the same time.

Of note, we should be aware of not all human CD4^+^ FOXP3^+^ T cells-expressing CD39. The proportion of this subset dramatically changes depending on ages (51) or diseases (52, 58, 59), indicating that CD39 expression to some degree might be of diagnostic interest. In addition, there is also a subset of CD4^+^ CD39^+^ T cells with lacking immunosuppressive function in peripheral blood (60). Actually, CD4^+^ CD39^+^ CD25^neg^ FOXP3^neg^ subset can be regarded as a reservoir of CD39^+^ Tregs since the former can be transformed into the latter upon staphylococcal enterotoxin B stimulation (61). Therefore, CD39 may be an indispensable chip to identify Tregs, but not absolutely unique.

### CD39 and γδ T Cell

Three decades have passed since the accidental but groundbreaking discovery of T cells expressing γ and δ chains in 1984 (62). But in the early stages, these immune cells were thought to be null and were suspiciously ill-represented in textbooks (62). The reasons for this negligence were majorly due to technical and conceptual difficulties in our understanding—how γδ T cells are generated in the thymus, which type of target structures they recognize, what the contributions they make to homeostasis, and when they take up action facing foreign pathogen or self antigen? γδ T cells belong to the non-conventional lymphocyte family though they can produce many cytokines of the same kind as αβ T cells such as IFN-γ, IL-17, and bear certain cell surface markers. But they are in possession of combination of cytotoxic function (63), follicular B helper function (64), antigen presentation function (65, 66), and regulatory functions (20, 67). Gδ T-APC, in particular, may represent a promising alternative to monocyte-derived dendritic cell in immunotherapy since γδ T-APC lack MHC restriction in antigen recognition and they are so easy to expand in large scale (68). Maybe it is not easy to categorize γδ T cells—are they regulatory cells, follicular B helper cells, cytotoxic cells, APC, or totipotent cell? This is indeed the case that γδ T cells share pleiotropic functions with conventional αβ T cells (69). As the research work goes further and more detailed, the classification of γδ T would be more reasonable. Maybe γδ T cells are composed of different subpopulations with different functions (see Table 1).

**Table 1:** Antigens stimulating different subsets of γδ T cells.

δ chain type	Paired γ chain type	Distribution	Antigens/restriction molecules	Reference
Vδ1	Vγ (several)	Skin, gut, reproductive tract, PB, spleen, liver	MICA, MICB, CD1c, CD1d, HLA-A24, HLA-A2, HLA-B27	(70–73)
Vδ2	Vγ9	PB	IPP, HMBPP, tetanus toxoid, Hsp60, Hsp65	(74–77)
Vδ3	Vγ9/3	PB, liver	CD1d	(78)
Vδ5	Vγ4	PB	EPCR	(79)

*PB, peripheral blood; MICA or MICB, MHC class I chain-related protein A or B; CD1c or CD1d, cluster of differentiation 1 isoforms; HLA, human leukocyte antigen; IPP, isopentenyl pyrophosphate; HMBPP, (E)-4-hydroxy-3-methyl-but-2-enyl pyrophosphate; Hsp, heat-shock protein; EPCR, endothelial protein C receptor*.

Human γδ TCR-expressing cells constitute 1–5% of total T cells in the peripheral blood but make up a major lymphoid subset in tissues such as the intestine and the dermis (80). Human Vδ1 T cells primarily reside in the dermis, gut epithelia, and are involved in maintaining epithelial tissue integrity (81). Vγ9Vδ2 (also termed as Vγ2Vδ2) T cells are a subset of γδ T cells in the peripheral circulation and play an indispensable role in host defenses against exogenous pathogens, immune surveillance of endogenous pathogenesis, and even homeostasis of the immune system. Recent researches have shown that CD277 plays a leading role (82) in Vγ9Vδ2 T activation and also CD39 on them is an important surface marker (8). Both of mice naïve and induced CD39^+^ γδ T cells expressed CD25 rather than FOXP3 nor CTLA-4, but have a stronger suppressive function *via* IL-10 (8). Recently, Hu et al. has identified a novel γδ-Treg subset exhibiting CD39 with stronger immunosuppressive activity than conventional CD4^+^ or CD8^+^ Tregs. More importantly, this CD39^+^ γδ-Tregs perform their suppressive function *via* the adenosine-induced pathway instead of TGF-β or IL-10 (20).

Herein, we highlight a recent study by Gruenbacher et al. who proved CD39 also hydrolyzes pAgs (phosphoantigens) which specifically activate Vγ9Vδ2 T cells (12) and thus revealed a previously unrecognized immune-regulatory role of CD39. By quantifying *Pi* in supernatant after incubating Vγ9Vδ2 T cells with different pAgs, the hydrolysis function of CD39 displays a cell dose-, substrate-, and time-dependent manner. Interestingly, geranylgeranyl diphosphate (GGPP, C_20_), which can also activate Vγ9Vδ2 T cells, resists CD39-mediated hydrolysis. Thus, GGPP, not other pAgs, would be exploited as a novel checkpoint for increasing the stability of ATP and pAgs (see Figure 2).

### CD39 and Th17 Cells

**Figure 3:**
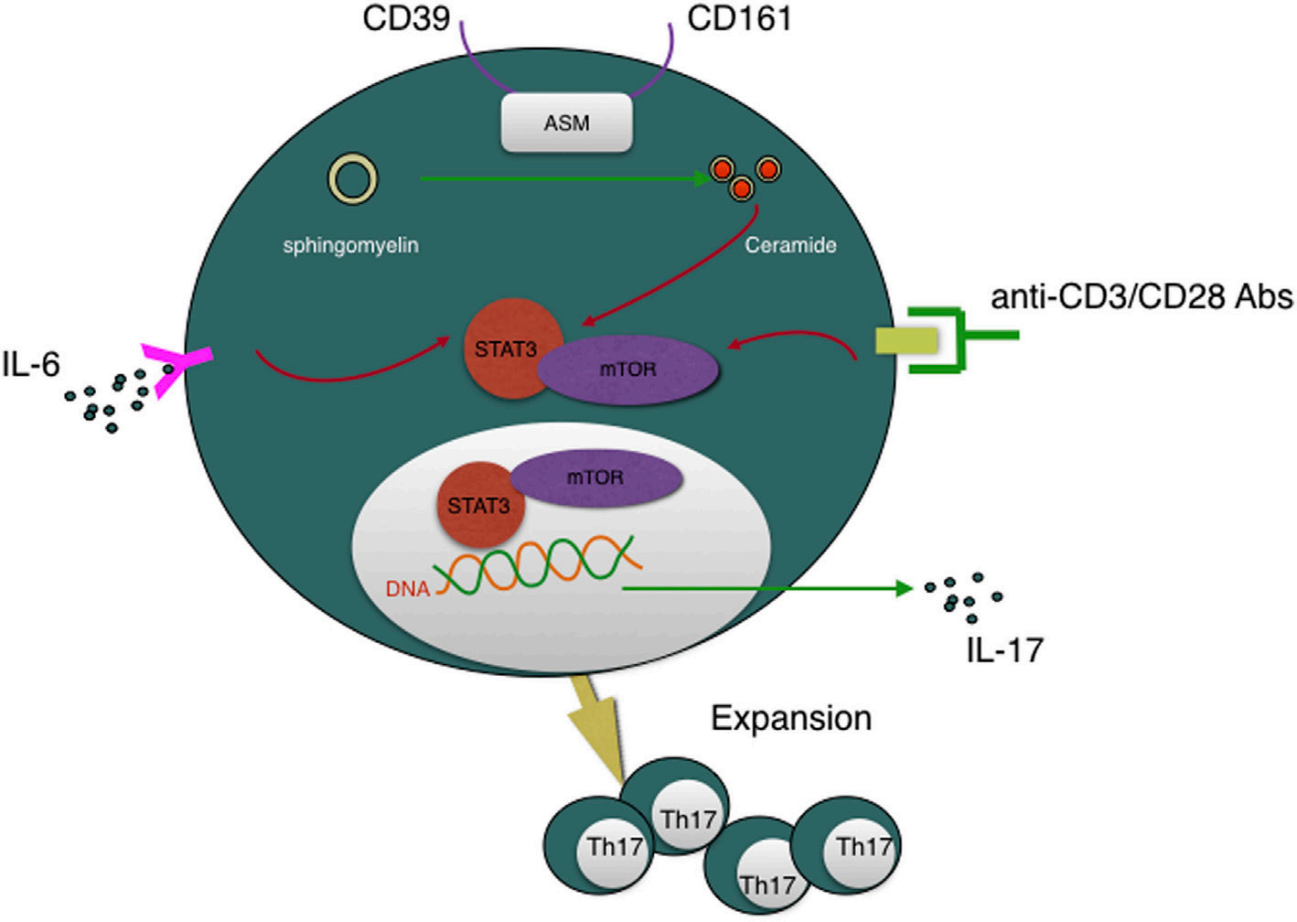
CD39 is involved in Th17 cells expansion and IL-17 secretion and, moreover, CD4^+^CD39^+^CD161^+^ T cells can be regarded as Th17 cells precursors. CD39, combined with CD161, can initiate acid sphingomyelinase enzymatic activity, subsequently, increase intracellular ceramide concentration, then impact STAT3 and mTOR signal transduction, which are essential for Th17 generation and IL-17 secretion.

Recently, Bai et al. reported that another novel findings that CD39 combined with CD161 drives Th17 cells expansion *via* acid sphingomyelinase (ASM) in Crohn’s disease patients (83). This conclusion, again, refresh our knowledge about CD39. Th17 cells are critical for host protective defense in adaptive immune responses and autoimmune diseases (84). But how CD4^+^ T cells polarize into Th17 cells subset is still a maze in addition to their lacking convincing surface marker. Bai et al. presented a novel notion that CD4^+^CD39^+^CD161^+^ can be used as Th17 precursors surface marker. Besides MHC/CD3/CD28 and IL-6/IL-6R pathway, CD39/CD161-ASM amplifies mTOR and STAT3 signals in another way, which eventually drive Th17 cells expansion and, subsequently, IL-17 secretion. Given the significance of Th17 cells, strategies to regulate CD39 and CD161 signaling may represent another novel approach to suppress Th17 responsiveness of inflammatory disease (see Figure 3).

### CD39 and Other Immune Cells

Indeed, CD39 was first described as a B lymphocyte activation marker (20, 85) and then be regarded as a T lymphocyte activation marker (86, 87). It is constitutively expressed on >90% of B cells, >90% of monocytes, 20–30% of CD4^+^ T cells (including memory T cells and Tregs), <5% of CD8^+^ T cells, and 2–5% of NK cells (6).

High-level expression of CD39 indicates CD8^+^ T cells terminally exhaustion especially in chronic viral infections, but it is not applicable to the CD8^+^ T cell compartment of healthy donors (88). In addition to be used as a marker of terminally exhausted CD8^+^ T Cells, CD39 also participates in the identification of CD8^+^CD39^+^CD26^−^ cells—a specific subset of CD8^+^ Tregs equipped with effective suppressive function *via* nicotinamide adenine dinucleotide phosphate oxidase 2 (NOX2) (89).

CD39 plays an indispensable part in DCs-driven CD4^+^ T cells activation and differentiation. For example, eATP activates the NLLRP3 inflammasome in DCs and this inflammasome is a prerequisite for the production of IL-1β and IL-18 (31). While the abovementioned cytokines are essential for Th17 and Th1 cells polarization, respectively (90). As for Th2 cells, Idzko et al. found that DCs in *Cd39* null mice showed weak capacity to induce Th2 immunity (91). They speculated that CD39-involved P2 receptors signaling might facilitate DCs to prime Th2 responses *in vivo*. Indeed, NLLRP3 is also supposed to promote Th2 cells polarization although this process is not by means of inflammasome form (92). Furthermore, accumulation of adenosine can impair the normal function of DCs, the so-called immune-suppressive regulatory DCs (93).

NK cells belonging to innate immune subset are characterized by mediating significant cytotoxicity, producing high levels of inflammatory cytokines and chemokines (22, 94, 95). Human NK cells can be modulated through activation of P2Y11R (20) and, therefore, CD39 can inhibit NK cells-mediated damage. There is indeed the case that CD39 deletion has been followed by the deficiency of IFN-γ by NK cells. In the context of tumor setting, expression of CD39 with consequently ATP degradation induced antitumor immune responses mediated by NK cells.

Human B cells have been reported to express CD39 and adenosine receptors (20). Figueiró et al. identified CD39^high^ B cells as the major contributor to Bregs (13). They also reported proliferation and functions of these CD39^high^ B cells are operated *via* adenosine generation and IL-10 secretion. Unlike previous research, the A1 and A2A adenosine receptors other than A3 adenosine receptor mainly mediate autocrine signaling in Bregs.

## The Role of CD39 in Inflammation

The purinergic system is a high-level delicate system adjusted to fine-tune immune cell functions. Varieties of cell types can release ATP or ADP from intracellular compartments into the extracellular spaces, besides apoptotic cells. eATP functions as a dangerous signal triggering activation of purinergic P2 receptors and hence subsequently a series of pro-inflammation responses (23, 96). This pro-inflammation cascade is terminated by conversion to suppressive molecule adenosine *via* CD39 and CD73. Similar to ATP, adenosine activates purinergic P1 receptors. During the process of inflammation, there are also several pathogenic microorganisms capable of inducing an adenosine-rich milieu, which favors them to escape host immune surveillance. By contrast, there is also evidence that overexpression of CD39 in mouse airways promotes bacteria-induced inflammation (97). The author speculated that CD39 might limit the desensitization of P2 receptors, which helps to promote airway inflammation in response to bacterial challenge. Consistent with the above results, CD39 expression and activity are elevated in chronic obstructive pulmonary disease (COPD) patients by quantifying CD39 expression and soluble ATPase activity in sputum and bronchoalveolar lavage fluid cells (98). Additionally, Tan et al. showed T cell-related CD39 expression is higher in acute exacerbations of COPD (AECOPD) patients than stable COPD and healthy controls (5). They prospected blocking CD39 would be a novel approach to the control of AECOPD, reducing the dependency on antibiotics.

In addition, some microorganisms themselves are equipped with high nucleotide metabolic versatility, which assist them with dissemination and invasion in the host (99). For example, Fan et al. reported that an ecto-5′-nucleotidase similar to human CD39 on cell surface of *Streptococcus sanguinis* contributes to its virulence (100). This analog takes effects by means of slowing the platelet aggregation response *in vitro* and reducing the accumulation of platelets on infected heart valves *in vivo* (100).

Hence, CD39, a pivotal enzyme between ATP and adenosine, is critical in preventing excessive P2R-mediated inflammation, but its function maybe turns detrimental for the appropriate clearance of apoptotic debris or by generating an immunosuppressive environment, which might promote the development or progression of cancer (101).

## The Role of CD39 in Tumor Immunity

Interactions between tumor cells and their immunological microenvironment are essential for the pathophysiology of tumor (102). CD39, the rate-limiting enzyme in the generation of immune-suppressive adenosine, undoubtedly plays a pivotal role in tumor progression. For example, CD39^+^ Tregs, mentioned above, inhibited NK cell antitumor immunity both *in vitro* and *in vivo* (20). Besides this, CD39 is expressed at significantly higher rates in tumor-infiltrating tissue such as ovarian, pancreatic, and testicular tumors, etc., in contrast to paired peritumoral tissue (55). Overexpression of CD39 was reported as a predictor of poor outcome for gastric cancer patient following radical resection (103). Additionally, CD39 expressed on Tregs was shown to play a permissive role in a mouse model of hepatic metastasis (104). Therefore, CD39 penetrates deep into to the modulation of tumor cell growth, differentiation, invasion, and migration (105, 106).

Importantly, after co-incubating these tumor cells with POM-1, the CD39 inhibitor, tumor-induced inhibition of CD4^+^ and CD8^+^ T cell proliferation was alleviated and CTL- or NK cell-mediated cytotoxicity increased. Consequently, treatment with a CD39 inhibitor or blocking antibody may become a promising strategy for ameliorating tumor cell-mediated immunosuppression. These data indicate CD39 to be a probable checkpoint in tumor immunotherapy and inhibiting CD39 may restore antitumor responses or boost the efficacies of other antitumor strategies. For example, treatment with ARL6715, another ectoATPase inhibitor, resulted in improving T cell responsiveness (107). The link between CD39 and tumor will become clearer since tumor-related research focused on CD39 is emerging in endless stream.

## Conclusion

The enzymatic activity of CD39, combined with CD73, plays a non-ignorable part in the shift from an ATP-mediated pro-inflammatory milieu to an immunosuppressive setting driven by adenosine. Some recent discovery about its new features such as hydrolyzing pAgs and catalyzing sphingomyelin suggest that CD39 have more unrecognized physical functions. Either way, in-depth study of CD39 will provide unique insights into the workings of immune network.

First, in basic research, CD39 can be considered as a novel bridge among immune cells. It is expressed on varieties of immune cells and there exists close relationship between CD39 and their functions. In the context of a subgroup, classification of immune cells based on CD39 may reflect their functions better. It is remarkable that CD39 is increasingly appreciated as a regulatory marker other than an activation marker. We can identify not only CD4^+^ Tregs but also CD8^+^ Tregs, Bregs, and γδ-Tregs based on CD39. As for its newly known feature about hydrolyzing pAgs, this may represent another mechanism by which CD4^+^ Tregs suppress γδ T cells. Besides “classical” functions such as cytokine production and cytotoxicity, recent studies suggest that γδ T cells are equipped with additional efficiencies such as regulatory activity (20) and—quite excitingly—“professional” antigen-presenting capacity (108). We should breathe calmly that it is absolutely worth a great number of costs on this fewer proportion.

Another emerging discovery about CD39 is closely related to CD161—they altogether modulate human Th17 cells responsiveness through alterations in ASM (10). CD39 and CD161 serve as potent surface markers of Th17 cells (10, 11), and furthermore, the latter has been identified as the top favorable pan-cancer prognostic molecule (109). There is still a long way to go to expound on the connection between CD39 and CD161. And what’s more, γδ T cells are also found to be a very important source of IL-17 in some disease models, particularly at early stage (110). Whether CD39/CD161-ASM-IL-17 chain also exists in γδ T cells has not been evaluated. Additionally, expression of CD39 is particularly associated with exhaustion of CD8^+^ T cells and identification of CD8^+^ Tregs, Bregs, both of which are being in the ascendant among immunological studies.

Second, the diagnostic potential of CD39 is only beginning to unfold. There have been already some studies proposing CD39 as a prognostic marker such as pancreatic cancer (105). In the setting of chronic lymphocytic leukemia, both CD4^+^ T cells and CD8^+^ T cells express high-level CD39 and furthermore, CD39 is associated with advanced disease stage (111). Besides, CD39 is also closely related to both Th17 cells and Tregs. In the context of inflammation or autoimmune diseases such as rheumatoid arthritis, Th17 cells stand for a pro-inflammatory subpopulation while Tregs have the antagonist effect. Hence, the Th17/Tregs balance can affect the outcome of immune responses. CD39, surprisingly, participates in the identification of both (see above). Fan et al. proposed identifying CD4^+^ T cell-derived CD161^+^CD39^+^ and CD39^+^CD73^+^ microparticles as new biomarkers for rheumatoid arthritis evaluation (11).

Finally, immunotherapy will be the direction of our long-term efforts and moreover, the possibly ultimate way out to inflammation and cancer. Immunomodulatory effects of CD39 brought a new dawn for immunotherapy. Since CD39 not only hydrolyzes eATP but also increases the concentration of anti-inflammatory adenosine, the administration of exogenous CD39, in nanoparticles or other forms may provide a new approach to limiting inflammation, which is likely to be more efficient than existing strategies aimed at blocking P2X. Alternatively, given the increased CD39 expression in apoptosis-prone T cells (51, 88), blocking CD39 with specific reagents might provide a novel checkpoint to induce a reaction cascade of protective responses in infection or tumor immunotherapy.

In summary, human immune system is a tight-knit social network while CD39 has correlation to various immune cells. It is now becoming increasingly appreciated that CD39 is a promising therapeutic target. Increasing or inhibiting CD39 can interfere with the abnormal pathophysiological process of disorders, especially inflammation and tumor. We are hopeful that the extensive impact of CD39 on the operation of immune response will be profoundly illustrated and its enormous therapeutic potential for a broad spectrum of diseases will be better exploited.

## Author Contributions

Each author has participated sufficiently in the work to take public responsibility for appropriate portions of the content.

## Conflict of Interest Statement

The authors declare that the research was conducted in the absence of any commercial or financial relationships that could be construed as a potential conflict of interest.
